# Effect of Narcissism, Psychopathy, and Machiavellianism on Entrepreneurial Intention—The Mediating of Entrepreneurial Self-Efficacy

**DOI:** 10.3389/fpsyg.2019.00360

**Published:** 2019-02-21

**Authors:** Wenqing Wu, Hongxin Wang, Chundong Zheng, Yenchun Jim Wu

**Affiliations:** ^1^College of Management and Economics, Tianjin University, Tianjin, China; ^2^Graduate Institute of Global Business and Strategy, National Taiwan Normal University, Taipei, Taiwan

**Keywords:** dark triad, narcissism, psychopathy, Machiavellianism, entrepreneurial self-efficacy, entrepreneurial intention

## Abstract

The driving factors behind the exploration and search for entrepreneurial intention (EI) are critical to entrepreneurship education and entrepreneurial practice. To reveal in depth the influence of personality traits on EI, our study introduces the opposite of proactive personality—the dark triad that consists of narcissism, psychopathy and Machiavellianism. Our study used the MBA students of Tianjin University as a sample to analyze the relationship between the dark triad, entrepreneurial self-efficacy (ESE) and EI. A total of 334 MBA students aged 24–47 years participated and the participation rate is 95.71%. The data collection was largely concentrated in the period from May 15 to June 5, 2018. From the overall perspective of the dark triad, the results show that the dark triad positively predicts EI, and ESE has a partial mediating effect on the dark triad and EI. From the perspective of the three members of the dark triad, the study found that narcissism/psychopathy has a negative effect on ESE and EI; narcissism/psychopathy has a non-linear effect on EI; Machiavellianism has a positive effect on ESE and EI; and ESE has a mediating effect on the three members of the dark triad and EI. In short, our research reveals that the three members of the dark triad have different effects on EI in different cultural contexts, and the research findings have certain reference value for further improvement of entrepreneurship education and entrepreneurial practice.

## Introduction

With the rapid development of science and technology, innovation, and entrepreneurial ability are increasingly becoming the main aspects of comprehensive national strength competition. Many global institutions credit entrepreneurship as a key driver of innovation and measurable economic development for their region (Montiel and Clark, 2018). Moreover, the importance of entrepreneurship to the economic, political and social environment of a country/region has been widely recognized, and there is a large amount of evidence related to its effect (Montiel and Clark, 2018). At the same time, an increasing number of studies show that entrepreneurship follows the formation of entrepreneurial intention (EI) (Zhang et al., 2014). In other words, EI is a strong predictor of entrepreneurial activity for individuals planning to establish a new business in the future (Obschonka et al., 2010). As a decisive factor in the creation of entrepreneurial behavior, EI has received extensive attention in the research field (Liñán et al., 2011).

Entrepreneurial intention is defined as a focused mentality that guides the individual’s attention and experience toward planned entrepreneurial behavior (Do and Dadvari, 2017). It refers to potentially enterprising individuals’ perception of business opportunities and deciding whether to create new businesses in the future (Thompson, 2009). Recently, the predictive effect of individual personality traits on EI has become a research focus in the field of entrepreneurial research. When talking about why people want to start a business, previous studies largely focused on proactive personality (Hu et al., 2018). Indeed, dark personality traits might also contribute to EI (Miller, 2015) because individuals tend to have multiple personalities, and dark personality traits can have a brighter side (Judge et al., 2009). Previous studies often overlooked the dark side of personality. However, scholars have recently noted that it is necessary to explore the influence of dark personality traits on EI (Denisi, 2015). Moreover, the dark triad has become a focus of the study of dark personality traits.

The dark triad, which consists of three sub-structures (narcissism, psychopathy, and Machiavellianism) (Paulhus and Williams, 2002), is a type of malicious mentality that primarily manifests as self-interest, aggressiveness, and ruthlessness (Mcdonald et al., 2012). Considering that psychopathy and antisocial personality disorder are similar but different in character, this study will emphasize and define psychopathy to distinguish them. Hare believes that the distinction between psychopathy and antisocial personality disorder is very important for clinicians and society (Hare, 1991; Gori et al., 2014). According to DSM-5, the basic characteristics of antisocial personality disorder are neglect and violation of the rights of others, manifested in irresponsibility, lack of self-accusation, pathological lying, lack of compassion, and aggressiveness (Gori et al., 2014; Gervasi et al., 2017). Psychopathy (or “primary psychopathy”) is a personality disorder characterized by lack of social norms, empathy and remorse. It is usually manifested by lack of anxiety, low withdrawal, impulse, guilt, manipulativeness and a persistent violation of social norms (Hare, 1991; Craparo et al., 2013; Gori et al., 2014).

Individuals with a high level of the dark triad tend to be oriented toward achievement (Jones and Figueredo, 2013). When faced with high challenges and uncertain situations, they are more confident and more outgoing than are others (Jonason et al., 2009). At the same time, entrepreneurship is a profession that requires a high degree of self-confidence and leadership and involves a high degree of uncertainty (Mathieu and St-Jean, 2013), which to some extent reflects the match between the dark triad and entrepreneurship. According to a recent study on the dark triad in the entrepreneurial field, the dark triad can positively influence the individual’s intention to start a risky endeavor, and the dark triad is one of the predictors of EI. For example, Kramer et al. (2011) believed that the dark triad had a positive effect on entrepreneurial innovation and achievement intentions. In addition, a high manifestation of the dark triad is related to the attractiveness of status and prestige, which might be reflected in entrepreneurial careers.

However, these empirical results and research conclusions are based on Western samples. Because China’s innovation environment system is incomplete and there are large cultural differences with the West, if we use China’s research samples for analysis, would doing so allow reaching a consistent conclusion? This question motivates our interest; thus, this study considers further exploration and verification in the context of China. Furthermore, studies suggest that the three members of the dark triad are distinguishable (Jonason and Webster, 2010). Other studies have argued that the relationship between the three members of the dark triad is moderately relevant, consistent, and mutually influential. Therefore, how do we treat the three members of the dark triad in the study? Should they be combined into one variable or interpreted as three independent variables to be analyzed separately (Adrian et al., 2013)? This question illustrates another issue that our study addresses.

In addition, the critical role of entrepreneurial self-efficacy (ESE) in entrepreneurial research has attracted the attention of scholars. ESE is a derivative concept of self-efficacy in the field of entrepreneurship (Chen et al., 1998). Entrepreneurship self-efficacy is defined as individuals’ belief in their ability to successfully start a business (Mcgee et al., 2009). Previous research on ESE has focused on whether ESE has a mediating role between proactive personality and EI. For example, Wang et al. (2015) analyzed the relationship between personality traits, ESE and EI through research on 295 students from an agricultural college in Central Taiwan. The study found that ESE has a mediating effect on positive personality traits (e.g., extraversion, openness, conscientiousness, and agreeableness) and EI.

Existing studies have performed research work on the mediating of ESE and have achieved remarkable results. Nevertheless, because human personality is often multi-faceted and complex, it is limiting to analyze the role of ESE only from a proactive personality perspective. Therefore, our study attempts to introduce the dark triad, analyzes the influence of the dark triad on ESE, and explores the mediating effect of ESE on the dark triad and EI. In summary, considering the existing research gaps and practical needs, our study attempts to explore the effect of the dark triad (including narcissism, psychopathy, and Machiavellianism) on EI and to analyze the mediating role of ESE.

The theoretical contribution of this study is primarily reflected in three aspects. First, this study addresses the gap in China’s research on EI and provides new insights into the relationship between the dark triad and EI. The influence of the dark triad on EI has obtained verifications and conclusions in the context of Western samples. However, in the Chinese context, there is a lack of corresponding research. Therefore, this study uses Chinese students as the sample objects to explore the relationship between the dark triad and EI. Second, this study explores its role in ESE and EI from the overall and local perspectives of the dark triad. This study uses the three members of the dark triad as a whole variable, but for comparison, they are included in the study as three independent predictors. Through comparative analysis, this study more comprehensively analyzes the effect of the three members of the dark triad on EI. Third, this study explores the influence path of the dark triad on EI from the perspective of mediating effect and provides new research ideas, laying the foundation for future research. Previous studies have focused on the mediating role of ESE in positive personalities and on EI using positive personalities (such as the five-factor model of personality) as pre-variables (Wang et al., 2015). However, our study uses the dark triad as a pre-variable, further explores the effect of ESE on EI, and further explores the influence mechanism of the dark triad on EI.

The content of this paper includes four aspects. First, we review the literature, use the life history theory as the theoretical basis, and propose the hypotheses of this study to be tested. Second, we use multiple regression analysis and use the MBA students of Tianjin University as a sample to verify the hypotheses that have been advanced. Third, we analyze and discuss the results of the empirical analysis, compare them with those from previous studies, and conjecture possible reasons for inconsistent research findings. Fourth, combined with the findings of this study, we propose the theoretical contributions and practical implications, summarize limitations and point to future research directions.

### Theoretical Background and Hypothesis Development

#### Dark Triad and EI

The dark triad is a socially malignant characteristic that is believed to be related to manipulative behaviors, selfishness, and exploitation (Jonason and Webster, 2010). From the perspective of social psychology, the dark triad is often considered unfavorable and abnormal (Do and Dadvari, 2017), but there is evidence that some dark-side traits might be beneficial in the business environment (Robie et al., 2008). Recent research has turned to the positive aspects of the dark triad.

Based on the life history theory, we try to understand the influence of the dark triad on EI. The theory holds that individuals choose behavioral strategies based on their environmental needs to maximize the likelihood of survival (Buss, 2009). If the future is uncertain and unpredictable, individuals with the dark triad often focus on meeting immediate needs and short-term relationships and adopting a fast-life strategy (Carter et al., 2015; Mannino and Faraci, 2017; Mannino et al., 2017). On the one hand, individuals with a high level of the dark triad are considered to adopt a fast-life strategy (Jonason et al., 2015), and they are more likely to try to initiate new venture creation (Hmieleski and Lerner, 2016). In other words, individuals high on the dark triad tend to regulate their behavior through fast-life styles, and they might choose to take risks such as creating start-ups (Jonason et al., 2010a). On the other hand, individuals with a high level of the dark triad are usually full of confidence, lack fear, disregard authority and the status quo, and adapt to operate in an unstructured and dynamic environment, which might make entrepreneurship an attractive career choice for these people (Jonason et al., 2010b).

##### Narcissism

The characteristics of narcissism are dominance, exhibitionism, and desire for sense of entitlement and superiority (Lee and Ashton, 2014). Compared with Machiavellianism and psychopathy, the highlighted contribution of narcissism to the dark triad is the feelings of entitlement and superiority toward others. Narcissists not only feel good about themselves but also feel that they are more worthy of respect from others (Jonason et al., 2013; Jones and Paulhus, 2014). Narcissists tend to be self-centered and constantly seek attention and admiration from others (Twenge et al., 2008), and they gain self-esteem, power, and status through the effective use of social relationships (Brunell et al., 2008). At the same time, narcissists lacks human values and desires to control others (Boddy, 2015), they are good at acquiring resources through their own charisma, letting other people adopt their plans (O’Reilly et al., 2014), and expecting others to accept their world view.

The influence of narcissism on EI is largely reflected in two aspects. On the one hand, narcissists tend to be leaders (or entrepreneurs). They usually have the ability to become dominant, a strong sense of control and grand self-awareness, and some qualities (e.g., charisma and strength) of narcissists overlap with the characteristics of leaders (O’Reilly et al., 2014). At the same time, entrepreneurship has become an admirable and individualized career choice that satisfies the psychological needs of narcissists, namely self-display and others’ admiration. Thus, individuals high in narcissism can immediately become leaders through entrepreneurship to live the fast life (Hmieleski and Lerner, 2016). On the other hand, narcissistic individuals prefer adventures. Individuals high in narcissism usually focus on gaining achievement and power and are not afraid of failure. Moreover, risky behavior can bring greater benefits, so unlike non-narcissistic individuals, they are more inclined to take risks (Jones and Figueredo, 2013) or to make higher-risk financial investments (Foster et al., 2011). The traits of narcissists that tend to be risky are highly relevant to participating in nascent start-ups and following fast-life strategies (Buss, 2009). In short, individuals high in narcissism consider themselves clever and attractive, they constantly seek admiration and superiority, and they tend to find leadership positions in an organization (Campbell and Campbell, 2009) and gain greater benefits and achievements through risk. Because entrepreneurship can better satisfy these motivational needs of narcissists, entrepreneurship can attract narcissistic individuals more than other career options might (Mathieu and St-Jean, 2013).

##### Psychopathy

Psychopathy refers to the inability to perceive, understand, or address emotions due to lack of emotional intelligence and empathy. Its main features are manipulativeness, deception, ruthlessness, and the pursuit of high excitement and stimulation (Akhtar et al., 2013; Crysel et al., 2013). Although psychopathy has negative connotations, individuals high in psychopathy are fully capable of successfully operating in daily life. Psychopathy might even motivate entrepreneurial intent, and there are good reasons to believe that psychopathy can be an important predictor of EI.

First, psychopaths are troubled by feelings about emotions. This defect causes them to be insensitive to loss or risk, which reduces or eliminates the risk suppression associated with fear of failure (Morgan and Sisak, 2015); thus, psychopaths dare to take risks. Although they cannot experience emotional empathy, this limitation does not prevent them from understanding the factors that drive people (that is, cognitive empathy) and makes them very capable of using other people to achieve their goals (Jonason and Krause, 2013). Second, individuals high in psychopathy hate social norms and are willing to oppose the status quo (Mathieu et al., 2013). They are good at concentrating and performing well in uncertain situations and are often drawn to exciting activities. Individuals high in psychopathy can avoid reporting to other people through entrepreneurship (Rindova et al., 2009) and can form their own corporate culture and norms. Third, the performance of psychopaths can often be related to positive attributions (Akhtar et al., 2013). Psychopaths are considered smart, charming and interesting people, and there are more “successful” psychopaths in top management (Mullins-Sweatt et al., 2010; Boddy, 2015). Successful psychopaths tend to balance impulsiveness and antisocial behavior with a higher sense of responsibility, thus playing a fairly successful role in the organization (Mullins-Nelson et al., 2006; Fennimore, 2017). Therefore, being high in psychopathy may help individuals become attractive leaders in start-ups. In short, the above analysis suggests that entrepreneurship provides a suitable and consistent opportunity for psychopaths to satisfy their personality traits; thus, psychopathy might contribute to entry into entrepreneurship.

##### Machiavellianism

Machiavellianism is a self-interested, deceptive, strategic, and manipulative personality trait (Zettler et al., 2011; Al Ain et al., 2013). Compared with narcissism and psychopathy, individuals high in Machiavellianism usually follow their own purpose, they are constantly pursuing the maximization of interests and have strong desire to control others (Zheng et al., 2017). They devote less emotion in interaction, primarily self-care, and rarely consider consequences to the people around them (Zettler et al., 2011). And they lack affinity and responsibility and tend to use persuasion and self-disclosure (Liu, 2008). In general, strong persuasiveness can help motivate others toward certain desirable outcomes (Do and Dadvari, 2017). In addition, individuals high in Machiavellianism have the desire to control and pursue status (Dahling et al., 2008), preferring to manipulate and utilize others to realize personal interests (Zheng et al., 2017).

Perhaps based on the above characteristics, individuals with Machiavellianism might be inclined to start a business. First, individuals high in Machiavellianism emphasize that the ends justify the means and have a strong imperative for money, power, and competition (Zettler and Solga, 2013). These people adopt short-term strategies that require immediate satisfaction and that are closely related to a fast-life approach (Jonason et al., 2017). Entrepreneurship might be one of the best methods for them to achieve these goals, because if entrepreneurship is successful, they will soon gain significant wealth and power. Machiavellian entrepreneurs can use the potential benefits (e.g., employment and taxation) provided by start-ups to prove the reasonableness of suspicious competitive means and behaviors, which are manifestations of short-term views and fast-life approaches (Hmieleski and Lerner, 2016). Second, Machiavellians have strong adaptability and can hide their true intentions and prejudices against others. Entrepreneurs with this personality show better strategic capabilities when making decisions (Ricciardi et al., 2018). This ability is very favorable for entering the entrepreneurial environment, because entrepreneurship is uncertain and unpredictable. Entrepreneurs are required to have a certain strategic vision and appropriate response capabilities. Third, Machiavellians tend to manipulate and use any necessary means to achieve their goals (Al Ain et al., 2013). They might make unethical decisions, and even win at the expense of others (Buckels et al., 2013). However, these behaviors can be useful in a new entrepreneurial environment (Klotz and Neubaum, 2016). Therefore, we propose the following:

H1: Dark triad has a significantly positive effect on EI.

H1a: Narcissism has a significantly positive effect on EI.

H1b: Psychopathy has a significantly positive effect on EI.

H1c: Machiavellianism has a significant positive effect on EI.

#### Dark Triad and ESE

The relationship between the dark triad and ESE has only received the attention and research of a few scholars. The dark triad is composed of three members (narcissism, psychopathy, and Machiavellianism), so we try to propose hypotheses from the overall and local perspectives of the dark triad. From the overall perspective of the dark triad, its three members are malicious mentalities or behaviors largely embodied in personal interests, aggressiveness, grandiosity, and callousness (Mcdonald et al., 2012). Some scholars have found that most entrepreneurs are characterized by manipulation, lack of compassion and obsession with status, which are very compatible with the characteristics of the dark triad. The scholars believe that these traits can play a vital role in self-efficacy (Zhao et al., 2010). Combining the above analysis of personality traits, we believe that these personality traits can enhance the belief that individuals with the dark triad would start a business in the future.

From the local perspective of the dark triad, narcissism emphasizes a high degree of self-recognition and desire to be concerned, although with some blind self-confidence, and highly narcissistic managers are good at creating and seizing opportunities (Do and Dadvari, 2017). Psychopathy is more manifested as indifference and low sensitivity to risk, but psychopaths can gain high social status and are considered smart, attractive and more efficient (Brunell et al., 2008). Machiavellianism is largely embodied in a strong desire for control and achievement, and individuals with high Machiavellianism are results-oriented and firmly pursue their goals (Zettler et al., 2011). Existing academic research has supported this view from a certain perspective. The researchers believe that entrepreneurs can be narcissistic and psychopathic, or they exhibit strong self-efficacy beliefs related to these personality traits (Marshall and Ojiako, 2015). The following sections demonstrate the relationship between the three members of the dark triad and ESE.

##### Narcissism

The main feature of narcissism is a sense of grandiosity, self-love and an expansive self-view (Foster and Campbell, 2007). Individuals with high levels of narcissism tend to have a high degree of self-recognition, are eager to receive attention, and are very good at creating and seizing opportunities (Do and Dadvari, 2017). Moreover, people high in narcissism have a strong motivation to pursue personal goals and are eager to self-improve and seek attention (O’Boyle, 2012). Generally speaking, narcissistic individuals have a full view of their abilities (Campbell et al., 2011); even when faced with opposing facts, it appears that individuals with high levels of narcissism believe they can do better than others will in the future (Mathieu and St-Jean, 2013). These qualities of narcissism encourage narcissists to have a higher confidence and belief in making something for themselves. For example, Brookes (2015) indicates that people with a higher level of narcissism tend to have a higher level of trust in their ability to achieve their goals, significantly promoting and actively predicting self-efficacy.

##### Psychopathy

Psychopathy is primarily characterized by deception, ruthlessness and a quest for stimuli (Crysel et al., 2013). Psychopathic people manifested more as being indifferent to others and less sensitive to risks, but they often gain high social status and are considered smart, attractive and more efficient (Do and Dadvari, 2017). On the one hand, psychopathic people have a certain maneuverability and ability to deceive, which helps them to achieve their goals through the ability and resources of others and can contribute to raise their belief in their ability to achieve entrepreneurial goals in the future. On the other hand, psychopathic people are less sensitive to risk (that is, even when they face the same risk, their perceived risk will be lower than it is to others). When facing an entrepreneurial environment full of uncertainty and risk, they are able to conduct objective analysis and address risk situations more calmly. These characteristics would enhance their ability to resolve entrepreneurial risks to a certain extent and improve their confidence in creating businesses in the future. In fact, psychopathic people can anticipate entrepreneurial careers that experience less debilitating anxiety and have a higher likelihood of obtaining positive returns, resulting in greater self-efficacy (Zhao et al., 2005). They are likely to weaken their concerns about their entrepreneurial career and have a stronger sense of control over the outcome. At the same time, they expect to receive positive returns and thus possess higher self-efficacy.

##### Machiavellianism

The main characteristics of Machiavellianism are the promotion of self-interest, deception, strategy and maneuverability (Al Ain et al., 2013). Individuals with high levels of Machiavellianism primarily show a strong desire for control and achievement, and they have a high degree of result orientation and of firm determination to pursue goals. On the one hand, individuals with Machiavellianism have a strong desire to control and a desire to pursue status (Dahling et al., 2008), preferring to use opportunities to maximize their own interests (Liu, 2008). They can adopt disreputable methods to achieve goals or maximize their own interests (Do and Dadvari, 2017). For example, they can use deceptive persuasion to guide and motivate others to the results they expect. This trait might raise the likelihood that individuals with Machiavellian would achieve their entrepreneurial goals. On the other hand, individuals with Machiavellianism generally have a higher demand for achievement. Individuals with higher achievement needs prefer to solve problems independently, like to take acceptable risks, and have a strong interest in the results of their efforts or decisions (Sesen, 2013). Moreover, greater demand for achievement will motivate individuals to courageously cope with challenging situations and pursue excellence (Sesen, 2013). To a certain extent, achievement needs could become a powerful driving force and an important belief for individuals with Machiavellianism. In other words, achievement needs are significantly positively correlated with ESE.

H2: Dark triad has a significantly positive effect on ESE.

H2a: Narcissism has a significantly positive effect on ESE.

H2b: Psychopathy has a significantly positive effect on ESE.

H2c: Machiavellianism has a significant positive effect on ESE.

### Mediating of ESE

The theory of planned behavior is an important theoretical basis for demonstrating the relationship between ESE and EI. The theory of planned behavior assumes that the intent of a particular target behavior depends upon perception (Prabhu et al., 2012). This type of perception in an entrepreneurial context includes a perception of one’s own skills and abilities, an attitude toward entrepreneurship, and a belief in starting a business in the future. In other words, the theory of planned behavior argues that attitudes, subjective norms, and perceptual control (self-efficacy) predict EI. In essence, ESE is primarily reflected in the recognition and perception of opportunities. When individuals have high opportunity recognition skills, they are more confident in perceived business opportunities and more accurately locate the products and services that consumers need; thus, they have stronger EI.

In fact, some studies have explored the relationship between ESE and EI through different perspectives and different samples. For example, Pittaway et al. (2011) studied self-efficacy in entrepreneurial situations and found that individuals have the belief that they can start a business when they have a high level of ESE. The research fully demonstrates that self-efficacy can predict an individual’s intention to start a new business.

Combining the arguments of Hypothesis 1 and Hypothesis 2 and the above analysis, we believe that the dark triad not only directly affects EI but also indirectly affects EI through ESE. Specifically, on the one hand, individuals with high levels of dark triad can have higher EI because they usually have a high appetite for risk and are keen to challenge uncertainty and risk in the field of entrepreneurship. More importantly, individuals with higher dark triad are confident in completing their business in the future and are good at debating and manipulating to achieve their entrepreneurial goals. On the other hand, individuals with high levels of dark triad usually have higher self-efficacy in entrepreneurship and further promote EI. Individuals with a high level of dark triad have a high degree of recognition for themselves and are eager to complete difficult and challenging things to receive attention. At the same time, their low sensitivity to risk motivates them to cope with risks in their entrepreneurial ventures. Their strong desire for control and achievement also motivate them to have a firm belief in starting a business in the future. Furthermore, these firm beliefs and high affirmation of themselves help to stimulate the creation of EI. Similarly, the three members of the dark triad also have direct and indirect effects on EI. Therefore, in our research model, we try to view ESE as the mediating mechanism linking the dark triad (including the three members of the dark triad) with EI.

H3: ESE will mediate the relationship between the dark triad and EI.

H3a: ESE will mediate the relationship between narcissism **and EI.**

H3b: ESE will mediate the relationship between psychopathy **and EI.**

H3c: ESE will mediate the relationship between Machiavellianism **and EI.**

## Materials and Methods

### Data Collection and Sample

This study conducted anonymous surveys on the dark triad, ESE, and EI of the MBA students at Tianjin University through a questionnaire survey. Multiple regression analysis was used to test whether the hypotheses were established. The reason for using this group as a sample for research is primarily based on (Hmieleski and Lerner, 2016). They believe that it is appropriate to use students studying business management as a sample to study the dark triad and EI because they represent business-oriented people generally. Moreover, they believe that doing so is unlikely to show omitted variable bias and endogeneity threats.

#### Questionnaire Design and Pilot Survey

A preliminary questionnaire was formed based on the existing mature scale. This study measures the dark triad, ESE, and EI. Given that each construct has been measured with a mature and validated scale, we adjust and integrate these mature scales as a final questionnaire for the study. Prior to the formal investigation, we randomly selected 20 volunteers to complete pre-test questionnaires. Based on their feedback and combined with the language expression habits in the Chinese context, the final questionnaire was established; it included 21 questions. The study and those questions did not involve any potential risks for participants. In this study, a seven-point Likert scale was used for the measurement of each item, ranging from 1 to 7, with “one” indicating that they “highly disagree” and “seven” indicating that they “highly agree.”

#### Sampling and Subjects

First, we selected the study sample. We obtained a list of all students who are currently enrolled in the MBA Education Center of Tianjin University, totaling 600 students. According to different grades, we selected 420 students as the subjects of the questionnaire survey through stratified sampling. Second, we performed data collection. The data collection was largely concentrated in the period from May 15 to June 5, 2018. Trained researchers informed participants that participation was voluntary and anonymous, and that the data collected were protected by applicable laws. Researchers distributed questionnaires and collected them during the students’ free time to ensure the quality of the answers to the questionnaire. Third, we initially screened and organized the data. A total of 402 questionnaires were returned and the participation rate is 95.71%. After eliminating 69 questionnaires that were incomplete and invalid, the final sample consisted of 334 valid questionnaires, with an effective rate of 83.08%. Among the valid questionnaires, 163 (48.80%) were completed by males and 171 (51.20%) by females. The age range is 24 to 47 years old, the mean age is 31.02, and the standard deviation (SD) is 4.088; 183 (54.79%) are married, and 151 (45.21%) are unmarried. Individuals whose relatives own businesses number 92 (27.54%), whereas 242 (72.46%) individuals have no relatives who own their own business.

### Measurement

Table 1 presents the measurement items for each construct. The items include narcissism, psychopathy, Machiavellianism, ESE, and EI.

**Table 1:** Measurement items and reliabilities.

Variables		Items	Factor loading	Alpha
The dark triad	Narcissism	I tend to want others to admire me.	0.704	0.801
		I tend to seek prestige or status.	0.742	
		I tend to expect special favors from others.	0.859	
		I tend to want others to pay attention to me.	0.860	
	Psychopathy	I tend to be callous or insensitive.	0.817	0.799
		I tend to be unconcerned with the morality of my actions.	0.855	
		I tend to lack remorse.	0.866	
		I tend to be cynical.	0.613	
	Machiavellianism	I tend to manipulate others to get my way.	0.704	0.823
		I have used deceit or lied to get my way.	0.845	
		I have used flattery to get my way.	0.850	
		I tend to exploit others toward my own end.	0.833	
Entrepreneurial self-efficacy		I am convinced that I can successfully discover new business opportunities.	0.919	0.921
		I am convinced that I can successfully create new products.	0.923	
		I am convinced that I can think creatively.	0.875	
		I am convinced that I can successfully commercialize ideas.	0.879	
Entrepreneurial intention		I am ready to do anything to be an entrepreneur.	0.829	0.937
		My professional goal is to become an entrepreneur.	0.878	
		I will make every effort to start and run my own firm.	0.923	
		I am determined to create a firm in the future.	0.920	
		I have the firm intention to start a firm someday.	0.914	


*This study measures all items with a seven-point Likert scale.*

#### Dark Triad

This study used the Dark Triad Dirty Dozen scale developed by Jonason and Webster (2010) to measure dark personality. The measurement scale consists of 12 items, and each personality characteristic of the dark triad is measured by four items. Based on the traits of narcissism (e.g., eager to be appreciated and expected to receive attention), the items for measuring narcissism include, for example, “I tend to want others to admire me,” and “I tend to want others to pay attention to me.” According to the prominent features of psychopathy, such as indifference and cynicism, the items that measure psychopathy include, for example, “I tend to lack remorse,” and “I tend to be unconcerned with the morality of my actions.” Combining the essential features of Machiavellianism, such as manipulating others and deceitfulness, measuring Machiavellianism’s items include, for example, “I have used deceit or lied to get my way,” and “I tend to manipulate others to get my way.” We use the arithmetic average of the four items for measuring narcissism, psychopathy, and Machiavellianism, respectively, as the final score of each construct. The higher the score is, the more prominent the corresponding personality characteristics are. Similarly, we use the arithmetic average of 12 items as the final score of the dark triad, and the higher the score, the higher is the level of the dark triad.

#### Entrepreneurial Self-Efficacy

We used the scale of Zhao et al. (2005) to measure ESE. ESE is a subjective measure of entrepreneurs’ ability to successfully accomplish something or achieve a certain goal in the future—for example, “I am convinced that I can successfully discover new business opportunities,” and “I am convinced that I can successfully create new products.” Calculating the arithmetic mean of the four items yields the final ESE score. A higher score indicates that the degree of ESE is higher.

#### Entrepreneurial Intention

For the measurement of EI, we use the measurement items developed by Liñán and Chen (2009), and the items have been verified. The measurement of entrepreneurial intent consists of five items that evaluate whether the research object has an intention to start a business—for example, “I will make every effort to start and run my own firm,” and “I am determined to create a firm in the future.” We calculate the arithmetic average for each item, and the higher the score, the more intense the EI.

#### Control Variable

Following existing studies on the factors that can affect EI combined with the study of Hmieleski and Lerner (2016), this study selects age, gender, marital status, and whether the family has an enterprise as a control variable. Older students usually have more work experience and broader social connections, which might cause them to consider starting their own business (Kautonen et al., 2015). With respect to existing studies, compared with women, men are more likely to start a business (Gupta et al., 2008). Given that entrepreneurship tends to have higher risks, married individuals might have a relatively low intention to start a business (Schiller and Crewson, 1997). The family environment usually has significant effects on individuals; therefore, if an individual’s relative owns a business, that individual might be more inclined to consider starting a business (Carr and Sequeira, 2007).

### Reliability and Validity

Through reliability analysis, we find that the Cronbach’s alpha coefficients of all constructs in this study are greater than 0.70 (as shown in Table 1), indicating that each construct’s measurement results showed good consistency (Cronbach, 1951). At the same time, these results show that the theoretical structure has good psychometric characteristics and meets the requirements of reliable internal consistency.

Based on factor analysis, a factor loading greater than 0.70 indicates that approximately one-half of the item’s variance can be attributed to constructs, which is a sign of construct validity (Fornell and Larcker, 1981). In this study, the factor loadings of the items are greater than 0.7 except for one item (as shown in Table 1), which fully demonstrates that the relationship between the items and the constructs is closer and that the questionnaire conforms to the aggregation validity requirement. In short, the reliability and validity of the overall questionnaire meet the requirements for further research.

## Results

Based on the Pearson correlation analysis, Table 2 presents descriptive statistics and correlations for variables. Observation shows that there is a significant correlation between the variables. The causal relationship between them will be verified through hierarchical regression analysis. At the same time, we note that the correlation coefficient between variables is less than 0.75 except for one, which can avoid the problem of multicollinearity to some extent.

**Table 2:** Mean, standard deviation, and correlation of study variable.

	Mean	*SD*	1	2	3	4	5	6	7	8	9
(1) Sex	1.510	0.501	1								
(2) Age	31.020	4.088	-0.195^**^	1							
(3) Married	1.450	0.498	0.213^**^	-0.478^**^	1						
(4) Family business	1.720	0.447	0.001	-0.168^**^	0.075	1					
(5) Narcissism	4.500	1.282	-0.200^**^	-0.058	-0.047	-0.076	1				
(6) Psychopathy	2.939	1.424	-0.143^**^	-0.160^**^	0.057	0.007	0.433^**^	1			
(7) Machiavellianism	3.180	1.452	-0.151^**^	-0.072	-0.018	-0.082	0.552^**^	0.756^**^	1		
(8) ESE	4.779	1.265	-0.199^**^	0.156^**^	-0.103	-0.205^**^	0.025	0.097	0.251^**^	1	
(9) EI	4.335	1.628	-0.200^**^	0.222^**^	-0.183^**^	-0.193^**^	0.058	0.166^**^	0.333^**^	0.680^**^	1


*N = 334. ^∗∗^*p* < 0.01.*

This section verifies each hypothesis through multiple regression analysis. When we formally test the hypothesis, we must use the variance inflation factor (VIF) to examine the possibility of multicollinearity. The results show that the maximum VIF value is 3.004, well below the threshold of 10; thus, there is no obvious multicollinearity problem (Neter et al., 1996). Table 3 presents the causal relationships among the dark triad, ESE, and EI.

**Table 3:** Results of multiple regression analysis.

	ESE		EI				
		
	Model 1	Model 2	Model 3	Model 4	Model 5	Model 6	Model 7
Sex	-0.457^***^	-0.394^**^	-0.443^**^	-0.351^*^	-0.116	-0.098	-0.032
Age	0.029	0.035^+^	0.058^*^	0.067^**^	0.028	0.036^*^	0.039^*^
Married	0.021	0.016	-0.204	-0.212	-0.239	-0.221	-0.224
Family business	-0.463^**^	-0.494^***^	-0.512^**^	-0.556^**^	-0.186	-0.163	-0.155
Dark triad		0.129^*^		0.290^***^			0.185^***^
Narcissism	-0.199^***^		-0.256^***^			-0.106^+^	
Psychopathy	-0.145^*^		-0.166^+^			-0.057	
Machiavellianism	0.392^***^		0.595^***^			0.299^***^	
ESE					0.832^***^	0.755^***^	0.810^***^
*R*^2^	0.173	0.100	0.239	0.147	0.491	0.527	0.508
ΔR^2^	0.155	0.086	0.223	0.133	0.483	0.515	0.499
*F*-Statistic	9.481^***^	7.069^***^	14.259^***^	10.977^***^	61.525^***^	44.021^***^	54.686^***^
Durbin–Watson statistic	1.762	1.752	1.847	1.860	2.172	2.131	2.154


*N = 334. ^+^*p* < 0.10; ^∗^*p* < 0.05; ^∗∗^*p* < 0.01; ^∗∗∗^*p* < 0.001.*

The first hypothesis predicts that the dark triad (H1) and the three personality traits of the dark triad, i.e., narcissism (H1a), psychopathy (H1b), and Machiavellianism (H1c), all have a significantly positive relationship to EI. Model 4 is used to verify the relationship between the dark triad and EI. In this model, we use EI as the dependent variable and use the dark triad as the independent variable. The results show that the dark triad has a significantly positive effect on EI (β = 0.290, *p* < 0.001); thus, H1 is verified. Model 3 is used to verify the relationship between the three personality traits of the dark triad and the EI. The model uses EI as the dependent variable and narcissism, psychopathy, and Machiavellianism as the independent variables. The results show that there is a significantly negative correlation between narcissism and EI (β = -0.256, *p* < 0.001), and there is a significantly negative correlation between psychopathy and EI (β = -0.166, *p* < 0.1). However, there is a significantly positive correlation between Machiavellianism and EI (β = 0.595, *p* < 0.001); therefore, H1c is supported, but H1a and H1b are not supported.

The second hypothesis predicts that the dark triad (H2) and the three personality traits of the dark triad, i.e., narcissism (H2a), psychopathy (H2b), and Machiavellianism (H2c), all have a significantly positive relationship to ESE. Model 2 is used to verify the relationship between the dark triad and ESE. The regression results show that there is a significantly positive correlation between the dark triad and ESE (β = 0.129, *p* < 0.05); therefore, H2 was verified. Model 1 was used to verify the relationship between the three personality traits of the dark triad and ESE. In this model, we use ESE as a dependent variable, and narcissism, psychopathy and Machiavellianism are used as independent variables. The results show that there is a significantly negative correlation between narcissism and ESE (β = -0.199, *p* < 0.001), and there is a significantly negative correlation between psychopathy and ESE (β = -0.145, *p* < 0.05). Machiavellianism is significantly positively correlated with ESE (β = 0.392, *p* < 0.001); thus, H2c was supported, but H2a and H2b are not supported. In other words, the higher the level of narcissism and psychopathy, the lower is the ESE, possibly because in the context of China, due to cultural differences, differences in value concepts, and differences in the entrepreneurial environment, the findings of this study are different from the existing conclusions.

The third hypothesis predicts that ESE has a mediating effect on the dark triad and EI (H3). At the same time, ESE has a mediating effect on narcissism and EI (H3a), ESE has a mediating effect on psychopathy and EI (H3b), and ESE has a mediating effect on Machiavellianism and EI (H3c). Models 2, 4, 5, and 7 jointly verify the mediating effects of ESE on the dark triad and EI. Model 7 uses the dark triad and ESE as independent variables and EI as a dependent variable. Models 2, 4, and 5 show that ESE has a mediating effect on the dark triad and EI. Models 4 and 7 show that ESE has a partial mediating effect on the dark triad and EI; therefore, H3 is supported. Models 1, 3, 5, and 6 jointly verify the mediating effects of ESE on the three personality traits of the dark triad and EI. Among these models, Model 6 uses ESE as a dependent variable and narcissism, psychopathy and Machiavellianism as independent variables. Models 1, 3, and 5 show that ESE has a mediating effect on the three personality traits of the dark triad and EI. Models 3 and 6 show that ESE has a partial mediating effect on narcissism and EI. Self-efficacy has a completely mediating effect on psychopathy and EI. ESE has a partial mediating effect on Machiavellianism and EI. Therefore, H3c is supported, but H3a and H3b are not supported. The assumption predicts that narcissism and psychopathy will positively affect ESE and thus promote EI, but the results show that narcissism and psychopathy negatively affect ESE, thereby inhibiting EI. In other words, although ESE has a mediating role between narcissism/psychopathy and EI, there is a negative conduction relationship between them; therefore, H3a and H3b are considered not supported.

Based on the research review of Smith et al. (2018), he emphasized that exploring the non-linear effects in dark triad has important theoretical value. Therefore, this paper attempts to expand research and analyze the non-linear relationship between the dark triad and EI in the field of entrepreneurial research (as shown in Table 4). We are pleasantly surprised to find that the square of narcissism has a significant positive impact on EI (β = 0.098, *p* < 0.01), that is, narcissism and EI is a U-shaped relationship, and the relationship between psychopathy and EI is a U shape (β = 0.069, *p* < 0.033). In other words, with the improvement of psychopathy/narcissism level, EI would gradually weaker, but when it exceeds a certain threshold, EI would gradually increase. This research did not find a non-linear relationship between Machiavellianism and EI (β = 0.038, *p* < 0.241).

**Table 4:** Examining the non-linear relationship between the dark triad and entrepreneurial intention.

	EI			
	
	Model 8	Model 9	Model 10	Model 11
Sex	-0.443^**^	-0.427^*^	-0.438^**^	-0.447^**^
Age	0.058^*^	0.050^*^	0.060^**^	0.059^*^
Married	-0.204	-0.234	-0.194	-0.194
Family business	-0.512^**^	-0.532^**^	-0.492^**^	-0.503^**^
Narcissism	-0.256^***^	-1.089^***^	-0.248^***^	-0.252^***^
Psychopathy	-0.166^+^	-0.165^+^	-0.635^**^	-0.181^*^
Machiavellianism	0.595^***^	0.586^***^	0.567^***^	0.328
Narcissism—quadratic effect		0.098^**^		
Psychopathy—quadratic effect			0.069^*^	
Machiavellianism—quadratic effect				0.038
*R*^2^	0.239	0.255	0.250	0.243
ΔR^2^	0.223	0.237	0.231	0.224
*F*-Statistic	14.259^***^	13.548^***^	13.191^***^	12.664^***^
Durbin–Watson statistic	1.847	1.850	1.849	1.849


*N = 334. ^+^*p* < 0.10; ^∗^*p* < 0.05; ^∗∗^*p* < 0.01; ^∗∗∗^*p* < 0.001.*

## Discussion

Research on the relationship between the dark triad and EI has been verified and discussed by Western scholars. However, personality is influenced by culture and environment. In the Chinese context, almost no studies analyze the relationship between the dark triad and EI. Our research conclusions show that there are similarities between the dark triad and EI in the context of Eastern and Western cultures, but there are also great differences and inconsistencies. This section discusses these points in detail in terms of the following aspects.

Some of this study’s findings are consistent with those found in Western scholars’ mainstream research. The dark triad has a significantly positive relationship with EI, which is consistent with the existing academic views in the West (Jonason et al., 2010b; Smith et al., 2018). The results prove that the dark triad has a certain predictive effect on EI. Although the dark triad is a socially malignant feature that is often thought to be related to manipulability and deception (Jonason and Webster, 2010), the dark triad might be advantageous in a business environment. Based on the life history theory, individuals with a high level of dark triad traits will be more inclined to choose a fast-life strategy and be better at taking risks (Jonason et al., 2015). Moreover, people with dark triad traits often lack a fear of risk, despise authority, and are good at operating successfully in an unpredictable environment. Thus, entrepreneurship could become the most attractive career choice for these people (Jonason et al., 2010b).

More importantly, other findings in this study differ from the conclusions in Western mainstream studies. The mainstream research of Western scholars indicates a positive correlation between narcissism and EI (Hmieleski and Lerner, 2016); that is, individuals with higher levels of narcissism are more likely to take risks (Mathieu and St-Jean, 2013) and have higher EI. There is a positive correlation between psychopathy and EI (Kramer et al., 2011), or there is no significant relationship (Hmieleski and Lerner, 2016); Western scholars found that there is no significant correlation between Machiavellianism and EI through empirical research (Kramer et al., 2011; Hmieleski and Lerner, 2016). However, this study found that narcissism/ psychopathy negatively affects EI. In other words, the higher the level of the individual’s narcissism and psychopathy, the lower the EI is. In addition, previous studies have shown that narcissism (Brookes, 2015) and psychopathy (Zhao et al., 2005) have a positive effect on ESE. The conclusion of this study is the opposite: narcissism/psychopathy and ESE have a significantly negative relationship.

Based on preliminary conjecture, we believe that these discrepancies might be due to differences in ethnic characteristics, differences in culture (or values) (Shinnar et al., 2012), and differences in the entrepreneurial environment (primarily entrepreneurial education) (Bae et al., 2014). First, in terms of ethnic characteristics, Chinese are more reserved and implicit, tend to rely on groups, and pursue stability. However, Westerners are more outward going, tend to be independent and free, and show strong aggressiveness. Second, in terms of cultural differences (or values), Chinese focus more on collective interests and advocate controlling their own desires. They oppose extreme individualism and heroism and often make personal interests subordinate to collective interests. In contrast, Westerners advocate personal will. They want to be appreciated and admired by others and have a strong desire to conquer. Third, in terms of entrepreneurial environment, China remains in the initial stages of creating an entrepreneurial environment. The relevant supporting business mechanisms and systems remain imperfect. Entrepreneurship education remains at the stage of exploration. However, Western countries have relatively perfect entrepreneurial support mechanisms and have a good entrepreneurial environment and atmosphere. These factors would more or less make the Chinese people’s EI lower, even when individuals show higher levels of narcissism and psychopathy.

## Conclusion

### Research Implications

The theoretical contribution of this study is largely reflected in three aspects. First, our study provides new insights into the relationship between the dark triad and EI in the Chinese context. Existing research findings show that the dark triad has a positive predictive effect on EI (Jonason et al., 2010a), and narcissism and psychopathy are both positive predictors of EI (Kramer et al., 2011; Hmieleski and Lerner, 2016). However, we found that although the dark triad has a positive predictive effect on EI, there is a significantly negative correlation between narcissism/psychopathy and EI in the Chinese context. We also found an interesting conclusion that narcissism/psychopathy and EI is a U-shaped relationship, which is the first time to analyze the non-linear relationship between the dark triad and EI through empirical research. In addition, existing studies have found that Machiavellianism has no significant effect on EI (Kramer et al., 2011; Hmieleski and Lerner, 2016), but we found that Machiavellianism has a significantly positive correlation with EI. In short, there is a certain difference between the findings of this study and the existing Western research conclusions. This study provides new insights for existing research and lays the foundation for further research.

Second, our study explores the roles of the dark triad on ESE and EI from the overall and local perspectives of the dark triad, which expands and enriches the existing research dimensions and research framework. This study uses the three members of the dark triad as a whole variable, and for comparison, they are included in the study as three independent predictors. Based on a comparative analysis, this study more comprehensively analyzes the effect of the three members of the dark triad on EI.

Third, our study explores the influence mechanism of the dark triad on EI through the perspective of mediating effect and addresses the gaps in existing research. Previous studies have focused on the mediating role of ESE in positive personalities and EI, using positive personalities (such as the five-factor model of personality) as pre-variables (Wang et al., 2015). However, our study uses the dark triad as a pre-variable, further explores the effect of ESE on EI, and further explores the influence mechanism of the dark triad on EI. The study finds that ESE has a mediating effect on the dark triad and EI. This study provides new ideas and perspectives for follow-up research.

### Implications for Practice

Our research findings have provided potential implications for business schools to conduct entrepreneurship education and government departments to build entrepreneurial systems. First, when conducting entrepreneurship education, business-school teachers should not only notice students’ proactive personalities but also focus on the understanding of students’ dark triad. The predictive effect of proactive personality on EI has been widely confirmed; the positive influence of the dark triad on EI has also received increasing attention. Therefore, in the specific practice, business-school teachers should teach students in accordance with their aptitude based on mastering their personality characteristics. In other words, according to the students’ personality traits, business-school teachers should formulate targeted entrepreneurship education programs, thereby giving full play to students’ personality advantages in entrepreneurship and innovation.

Second, in the Chinese context, business-school teachers should conduct appropriate intervention and guidance for students with a high level of the dark triad. From the overall characteristics of the dark triad, individuals with a dark triad are more inclined to start a business. Business-school teachers should give special attention to these students and provide entrepreneurial encouragement and assistance when they can. Because they are less fearful than ordinary students are and tend to take risks, they have more intense EI. Most importantly, they dare to use entrepreneurship as their career choice. Based on the local characteristics of the dark triad, if students are high in narcissism and psychopathy, business-school teachers should use certain measures and education methods to intervene and to reduce their level of narcissism and psychopathy, which will help to enhance their EI.

Third, when undertaking entrepreneurship education and building an entrepreneurial system, relevant departments must combine Chinese cultural characteristics and environmental characteristics. In view of the great differences between the findings of this study and the Western mainstream research conclusions, we preliminarily speculated that these differences are due to cultural differences and differences in values. Thus, business-school teachers should consider the differences between Eastern and Western cultures when conducting entrepreneurship education and combine the personality traits of local students to teach them in accordance with their aptitude. At the same time, the relevant government departments should fully integrate the Chinese cultural tradition and characteristics when constructing the entrepreneurial system and give full play to the promotion and leading role of the entrepreneurial system to potential entrepreneurs.

### Limitations and Suggestions for Future Research

We now summarize a few limitations of the current study and provide suggestions for future research. First, the findings of our study are different from those of existing studies. However, the factors behind these differences have not been explored. The findings of this study are different from the Western research findings. We tentatively believe that these differences might be due to differences in the research context (e.g., our study is primarily based on the Chinese context) and sample selection. We suspect that these differences might be due to differences in ethnic characteristics, cultural differences (or values), and differences in the entrepreneurial environment (e.g., entrepreneurial education). However, due to the limited purpose of our study, we did not discuss the reasons behind the differences in detail. Therefore, future research might further explore the key factors that lead to the differences by conducting horizontal comparative analysis of the East–West samples.

Second, our study did not consider the effect of the combination of proactive personality and the dark triad on EI. Existing studies not only find that a proactive personality has an important influence on EI but also show that the dark triad has important effects on EI. Because individuals often have complex personality dimensions, to more fully explore the influencing factors of personality traits on EI, future research might consider introducing proactive personality and the dark triad into the research model at the same time to analyze their interaction (Smith et al., 2018) and explore their effect on EI.

Third, the measurement method adopted in this study have displayed adequate psychometric properties in existing research, but self-report questionnaires can cause the research results to be affected by common method variance (Do and Dadvari, 2017), social desirability and cultural bias (Granieri et al., 2017). Our study controls the effect of common method variance by adjusting the order of items in the questionnaire and reducing the research object’s guess concerning the purpose of the measurement. Moreover, the study’s low-stakes setting is sufficient to overcome the problem of faking-good (i.e., participants have no incentive to falsify their response) (Akhtar et al., 2013). In addition, considering time perception has important influence on human behavior and choice (Mannino and Caronia, 2017; Mannino et al., 2017), future studies might consider measuring at different time points (e.g., re-answering the same question at intervals). Future studies also can try different methods of collecting data on the same research object (e.g., online questionnaires, oﬄine scales, and interviews). Especially for the measurement of psychopathy, due to psychopaths use manipulation, the use of a structured or semi-structured interview (e.g., PCL-R) is more adequate. Therefore, future studies can adopt the above measures to more objectively and comprehensively reflect the personality characteristics of the surveyed people in future studies, thus providing more-powerful support for the research results.

Fourthly, this study only involves MBA students from Tianjin University as volunteers to participate in the survey, which limits the generalizability of the results. Future studies ought to expand the sample to study individuals in different cultural backgrounds. In addition, Hare believes that the clinical concept of psychopathy is far more salient and robust than many researchers have imagined (Hare, 1980). Therefore, inspired by the research methods of Lo Coco et al. (2018), future research can analyze the influence mechanism of dark triad (Machiavellianism, psychopathy, narcissism) on EI through comparative analysis of clinical group samples and non-clinical group samples.

## Ethics Statement

Ethics approval for this research was not required as per institutional and national guidelines. Consent from all research participants was obtained by virtue of survey completion.

## Author Contributions

All authors listed have made a substantial, direct and intellectual contribution to the work, and approved it for publication.

## Conflict of Interest Statement

The authors declare that the research was conducted in the absence of any commercial or financial relationships that could be construed as a potential conflict of interest.
